# Water Supply and Waste

**Published:** 1893-01-14

**Authors:** 


### Water Supply and Waste.

This is an age that believes in water and plenty of it. Only a few ventured to protest when it was first proposed to draw on Loch Katrine and Thirlmere for the supply of two of our great towns, and they were cried down as insanitary sentimentalists. Truth to say, the lakes seem none the poorer for their loss and the towns are all the richer. They have, at least, the possibility of cleanliness given to them; one might desire that they used it more. But we are, we Britons, taking us on the whole, a cleanly people—cleanly, at least, compared with some of our neighbours—and because of this we are enabled to escape more easily than they from epidemics of all kinds. May it be so with cholera! The water supply of London is not, indeed, as large as one might desire; Edinburgh has been agitating for an increase for years; Birmingham promises itself an ample sufficiency in the immediate future; Glasgow and Manchester are already well supplied. Even small towns and villages have an abundance of which the last generation never dreamed; and the present writer was surprised not long ago to find that Tomintoul, the highest inhabited village in the Highlands, if not in the British Isles, was blest with gravitation water, brought from the neighbouring hills.

In short, we have almost everywhere in Britain a very fair supply of water, and proposals for increasing this usually meet with a ready consent from the legislature. This is as it should be; but there is another side to the question. Heavily-burdened ratepayers are not as sympathetic on the subject as amiable M.P.'s, and grumble about having already an ample supply of water if it were not wasted. It must be owned that these grumblers take up a rather ungracious attitude, and if they venture to air their sentiments in the columns of the local papers they draw upon themselves endless abuse. They are described as the champions of dirt and disease, as driving the poor man to the public-house and beer because he cannot get a drink of decent water; as compelling the children of the poor to go unwashed, and breaking the heart of the conscientious housewife by withholding from her the means of making her home all that a home should be. But the objectors may now have the satisfaction of knowing that all their reasonable complaints are endorsed by the *Sanitary Journal*, the organ of the Sanitary Association of Scotland, a body which it would be unreasonable to suppose hostile to cleanliness and health.

Water is wasted—foolishly, thoughtlessly, and wilfully. The lavish use of water is not inevitably waste. The woman who takes three times as much water to wash her floor or her clothes as her neighbour does, will, in all probability, wash them three times as well. You very rarely find a good worker in any department who does not demand a sufficiency of material. The man who takes his tub every morning cannot, except in times of exceptional drought, be accounted extravagant, though we recall one prominent Edinburgh citizen who boasted, when agitating for a larger water supply, that so determined was he not to stint his neighbours that he had not taken a bath for six months. It is to be feared that he got more derision than respect by way of recompense. An occasional over-indulgence in water, externally or internally, is one of the failings that leans to virtue's side, and we would not be too hard on it. But you will hardly go through a village street without seeing a pump left streaming by some careless water-drawer, or worked for no end but mischief by some thoughtless urchins. In towns the inconsiderate individual who wishes to flush his drains, and therefore fixes a tap or handle so that uncounted gallons may run away while he goes off to his work, oblivious; the same individual when, in time of frost, leaves all the taps in his house running at the full all through the night; the servant, known in almost every kitchen, who turns off the water so carelessly that a continuous dribble falls into the sink, powerless to cleanse it but sufficient to waste; these are the people of whom their fellow-citizens have the right to complain. They do no good, and they lessen the quantity of water available for others. Water, like other things, has to be paid for, and when it has to be brought from any great distance it is an expensive commodity. We pay for it, indeed, but in such a fashion that many people do not realise its cost. The charge is a matter of guesswork at the best. The fashion of charging the water-rate in proportion to the rental has the merit of expediency, but it can make no pretence to being a *quid pro quo*, accurate payment for value received. A small family may live in as costly a house as a large one; but it is to be assumed—one might say, it is to be hoped—that the latter will consume more water. Therefore one household pays for more than it consumes, another, perhaps, for less. To remedy this injustice, it has been proposed to have water meters, as we have gas meters, and pay for water as we do for gas, according to the amount consumed. Some of these meters have been tried, and they show very startling contrasts in the quantity of water used by next-door neighbours, people who, in all outward semblance, are subject to the same needs and possess the same habits. But one family uses less than ten gallons a day and the other nearly fifty. Hospitals are among the largest consumers, and the economists sometimes class them with the extravagant. The record of Edinburgh Infirmary goes up into hundreds of gallons, and so in all probability do many others; but when one thinks of the extreme importance of absolute cleanliness in everything connected with the sick, one must exonerate the hospitals from blame. But with all the advantages which water meters may possess, they may tend to an economy which is not desirable. We are all scrupulously careful of the gas for a week or two after the quarter's bill comes in; that is human nature. But in turning down the gas in the hall and allowing only one burner in the sitting-room, in singing "Early to bed" with wearisome persistence, for the sake of economising in the extra hour or two which we take from our books and give to slumber, though we may inconvenience ourselves and those of our household, we do no serious harm to anyone. Not so when we begin to stint in the use of water. The inconvenience may be less keenly felt, but it is more serious in its results. Water is the foe of dirt and dirt is the friend and kinsman of disease. But disease, once it has come, does not confine itself to its lawful prey; the innocent suffer with the guilty; the dirty infect the clean. Under the impression that water is public property, people will use it freely who would stint at every turn if they had to pay for it at so much a gallon. Alas that it should be so! But human nature is so constituted that it likes to get anything from the Government, or the public, or any other vague noun of multitude, not realising that the public is no more than the aggregate of its rate-paying self. Since, then, people will use water only when they imagine they get it for nothing, let us leave them to their delusion. After all, there are more who use too little water than too much. Let them be instructed in the error of their ways. At the same time, however, it is well to know how water can be used to no profit, so that we may not, through ignorance or thoughtlessness, lessen our own and our neighbours' share of one of earth's greatest boons.

